# Psychosocial impact of Covid-19 outbreak on Italian asthmatic children and their mothers in a post lockdown scenario

**DOI:** 10.1038/s41598-021-88152-4

**Published:** 2021-04-28

**Authors:** Daniela Di Riso, Silvia Spaggiari, Elena Cambrisi, Valentina Ferraro, Silvia Carraro, Stefania Zanconato

**Affiliations:** 1 Department of Developmental Psychology and Socialization, University of Padova, 35131 Padova, Italy; 2 Department of Womenʼs and Childrenʼs Health, University of Padova, 35128 Padova, Italy

**Keywords:** Psychology, Diseases, Respiratory tract diseases, Asthma, Paediatric research

## Abstract

Italy was the first European country to fight the Covid-19 outbreak. To limit the transmission of the virus, the Italian Government imposed strict domestic quarantine policies and temporary closure of non-essential businesses and schools from March 10th,2020. Although more and more literature is exploring the impact of the pandemic on non-referred children and families, only a few studies are focused on the psychosocial impact of Covid-19 in chronically ill children and their caregivers. The present study investigates asthma control and children and mothers’ psychological functioning (i.e.: psychological well-being, fear of contagion, and mothers’ Covid-19 related fears) in 45 asthmatic children aged 7-to-14, compared to a control sample. The subjects were administered an online survey after the lockdown (from 28th May to 23rd August 2020). The analysis shows that asthmatic children presented higher concern in relation to contagion, however, no difference in psychological functioning was displayed between the two cohorts. Mothers reported more Covid-19 related fears, and greater worries according to the resumption of their children’s activities. Moreover, they indicated a global worsening of their psychological well-being during the lockdown. Furthermore, regarding the clinical sample, the multivariate regression model showed that a worsening of mothers' psychological and children’s physical well-being was associated with a worsening of children’s psychological well-being during the lockdown. The results of this study indicate that mothers of asthmatic children can be more prone to experience psychological fatigue in a pandemic scenario. Special programs should be developed to support caregivers of chronically ill children.

## Introduction

By the end of January 2020, the World Health Organization declared great concern about some cases of COVID-19 registered in China^1^. Italy was the first country outside of Asia and the first in Europe where verified coronavirus cases appeared. On March 9th, the Italian Government imposed a national lockdown to limit the spread of COVID-19, imposing strict domestic quarantine policies, temporary closure of non-essential businesses and schools of every order and degree (Phase 1). These mandatory limitations changed over time, with a prudent reopening of selected commercial services (Phase 2—from May 4th to June 14th) and softening movement restrictions (from May 18th). Phase 3 started on June 15th: the Government authorized the opening of all the activities, including recreational centres for children and adolescents, and released all the mobility restrictions. Many studies focused on Italian children's physical and psychological functioning, reporting poor sleep quality and increasing emotional symptoms, such as sadness or restlessness during Phase 1^2–4^. Moreover, the extreme reduction of in-person contact with peers is reported to negatively affect children's perceived psychological well-being^5^. Concerning chronically ill children, literature has stressed the importance to assure continuous treatment also in a home-confinement scenario, providing guidelines for specific chronic conditions (e.g. cancer^6^, cystic fibrosis^7^). Very few studies explored the psychological effects of the Covid-19 pandemic on chronically ill children and their families^8–10^.

Asthma is the most common chronic disease among children. In the initial phases of the pandemic, patients with chronic lung diseases, including moderate-severe asthma and allergy were considered at highrisk of developing severe Covid-19 symptoms. Nonetheless, the association between asthma and COVID-19 was unclear and still debated at the beginning of the pandemic^11–14^. According to epidemiological studies, it seems that there has been a significant decrease in pediatric asthma admissions during the home-confinement^15^. However, literature reported that children with milder symptoms of asthma could be more at risk of worsening their symptomatology, due to the priority given to severe respiratory diseases, the greater exposure to indoor noxious environments, or the decrease of medical in-person exams in COVID-19 scenario^16–18^. Therefore, considering that asthma exacerbations in childhood are often related to viral infections, asthmatic children and their parents could be more worried about having worse outcomes if contracting COVID-19 and undertreating of eventual SARS-CoV-2 related respiratory symptoms^19^**.** Furthermore, the similarities between asthma and Covid-19 symptoms may represent another reason of concern for asthmatic children’s mothers, also considering the gradual reopening of activities in a post lockdown scenario^16^. As far as we know, no previous studies explored the psychological impact of Coronavirus outbreak on asthmatic pediatric patients and their caregivers. More recent research suggests that the risk of having respiratory attacks is associated with higher separation anxiety symptoms in asthmatic children, which may be amplified in a post lockdown scenario^20^.

Moreover, literature mentions that mothers who report general psychosocial well-being are more prone to handle the management of their children’s asthma and to contain their eventual concerns about the disease^21^.

The general aim of the present paper was to assess the psychological functioning and the Covid-19 related fears in a group of asthmatic children and their mothers in a post lockdown period, to evaluate the effects of home-confinement experiences (Phase 1) in a reopening scenario (Phase 2).

## Methods

### Subjects

Sixty-four asthmatic children aged 7 to 14 years and their mothers were recruited among those regularly followed at the Unit of Pediatric Allergy and Respiratory medicine of the Women and Children’s Health Department (University of Padova, Italy). All asthmatic children with annual control visit scheduled between May and July 2020 and in line with the exclusion criteria, were selected and the study was presented to their mothers. This time frame was chosen to retrieve information immediately after the lockdown imposed by the Covid-19 pandemic. Nineteen mothers did not agree to participate in this research. Therefore 45 asthmatic children and their mothers were enrolled (70.3% response rate). Exclusion criteria were comorbidity with psychiatric or other chronic diseases, poor comprehension of the Italian language, severe asthma treated with biological drugs. Forty-one healthy children matched for age and gender to the clinical sample and their mothers were recruited as a control sample. As schools were closed due to the Covid-19 pandemic, the study was introduced to families recruited through snowball sampling. Exclusion criteria were the presence of asthma or other chronic and psychiatric diseases, and poor comprehension of the Italian language. Mothers of the two groups did not differ according to age, schooling, occupation, and working situation during and after home-confinement as the Student’s t-tests and χ^2^ tests demonstrated (*p* > 0.05).

### Procedure

A survey online was administered from 28th May to 23rd August 2020. The study was introduced to parents by pediatric pulmonologists during a check-up phone call scheduled in April–May 2020 for a reassessment of asthma. Families who agreed to participate were sent an email including the link for the survey and an alphanumeric code to insert at the beginning of the form. Children completed their part immediately after their mothers’ one and each section took about 20 min. To begin with, mothers were asked about their socio-demographic characteristics (for example gender, age, schooling, employment, and working situation during and after home-confinement). Secondly, mothers and children filled a survey created ad hoc for the study which included questions related to the COVID-19 pandemic (e.g., how much they felt worried about the COVID-19 infection), perceived change in physical and psychological well-being comparing a pre and post-COVID-19 period, and, specifically for children, questions about contacts with friends during and after the home confinement. Lastly, they both had to complete standardized self-report questionnaires, assessing respectively general well-being (General Health Questionnaire, GHQ-12)^22,23^ and COVID-19 related fears (Multidimensional Assessment of COVID-19 – Related Fears, MAC-RF)^24^ for mothers, and psychological adjustment (Strengths and Difficulties Questionnaire, SDQ)^25,26^, and separation anxiety (Separation Anxiety factor of the Spence Children Anxiety Scale, SCAS-SAD)^27–29^ for children.

The procedure for the control sample was the same as for the clinical sample. The surveys were almost identical, except for the items regarding asthma.

Besides, the medical team provided clinical data regarding asthma control (Asthma Control Test, ACT^30,31^, Global Initiative for Asthma (GINA) score^32^) and severity (GINA therapeutic steps^32^), obtained during asthma reassessment in April–May 2020.

The project was approved by the Institutional Ethical Committee of Padua (Prot. n. 3671). The research project was performed in accordance with the Ethical and Deontological codes of Italian Psychologists. A detailed informant consent needed to be signed to join the survey, both from the mother and the child if 12 years old or older. 45 mothers gave their consent for their own study participation. As to the 45 children, informed consent was obtained from a parent or legal guardian No reward was offered for enrollment.

### Measures

#### Children asthma control and severity

Asthma Control Test (ACT)^30,31^ is a validated screening tool completed by children that address asthma control. The version for children younger than 12 years old includes 4 questions for the child (like “Do you cough because of your asthma?”) and 3 questions for the parents (like “During the last 4 weeks, how many days did your child have any daytime asthma symptoms?”) rated respectively on a 4 and 6-point Likert scale. Whereas, the version for children over 12 years old is made up of 5 questions about activity limitation, shortness of breath, night-time symptoms, use of rescue medications, and patient’s overall rating of asthma control over the previous four weeks. The questions are rated on a 5-point Likert scale. Higher scores indicate better-controlled asthma.

Global Initiative for Asthma (GINA) score. The Global Initiative for Asthma (GINA) guidelines^32^ classify asthma control through medical staff investigation of 5 factors: daytime symptoms, night awakening, need for relievers, limitation to physical activity, and spirometry parameters. Based on the GINA guidelines^32^, three levels of asthma control were identified: well-controlled (score 0), partially controlled (scores 1 and 2), uncontrolled (scores 3 and 4).

GINA therapeutic steps, based on the GINA guidelines^32^, classify asthma severity according to the pharmacological regimen needed (types of medicine, dosages, and frequencies of administration) into 5 therapeutic steps: 1 and 2 for mild asthma, 3 and 4 for moderate asthma, and 5 for severe asthma.

#### Children’s psychological functioning

Strengths and Difficulties Questionnaire (SDQ)^25,26^. The questionnaire is a validated behavioral screening tool composed of 25 items, rated on a 3-points Likert scale (from 0 = not true to 2 = certainly true) and 5 subscales: emotional symptoms, conduct problems, hyperactivity and inattention, peer problems, and prosocial behaviors. By adding the first four scales, a total difficulties score can be calculated. Higher scores indicate more problematic behavioral traits^26^. In this study, Cronbach’s α for the total score (TDS), the internalizing symptoms scale (INT), the externalizing symptoms scale (EXT), and the prosocial behaviors scale (PROS) were respectively α(TDS) = 0.651, α (INT) = 0.490, α(EXT) = 0.636, and α(PROS) = 0.305.

Separation anxiety factor of the Spence Children Anxiety Scale (SCAS-SAD)^27–29^. The separation anxiety factor is one of the 6 which compose the SCAS questionnaire (the other factors are: panic and agoraphobia, fears of physical injury, social phobia, obsessive–compulsive problems, and generalized anxiety/overanxious symptoms); for this study, only this factor was used. It includes 7 items on a 4-point Likert scale (from 0 = never to 3 = always) that assess separation anxiety symptoms. Higher scores indicate higher levels of separation anxiety symptoms. In the present study, Cronbach’s α for the separation anxiety factor was α(SCAS-SAD) = 0.731.

#### Mothers’ psychological functioning

General Health Questionnaire (GHQ-12)^22,23^. This questionnaire allows evaluating the presence of minor psychological disorder in primary care settings through the administration of 12 items rated on a 4-point Likert scale (from 1 = more than usual to 3 = much less than usual). GHQ-12 total score can be classified in three ranges: no presence of difficulties (lower scores), presence of minor difficulties, and presence of important difficulties (higher scores) which may indicate the need for professional intervention. In this study, Cronbach’s α was α(GHQ-12) = 0.701.

Multidimensional Assessment of COVID-19—Related Fears (MAC-RF)^24^. The questionnaire is composed of 8 items that investigate 8 types of COVID-19 related fears: fear of the body, fear for the body, fear of others, fear for others, fear of knowing, fear of not knowing, fear of action, fear of inaction. Items are grouped into 4 subscales: fears related to the body, fears related to meaningful relationships, difficulties in cognitive monitoring of concerns, and behavioral difficulties related to fear. Respondents rate all 8 items on a 5-point Likert scale (from 0 = very unlike me to 4 = very like me). By adding all items’ rates, a total score is obtained. The higher it is, the stronger Covid-19 related fears are. In this study, Cronbach’s α was α(MAC-RF) = 0.767.

### Data analysis

Student’s T-test was performed to assess the differences between mothers and children of the two samples in standardized questionnaires’ scores (GHQ-12 and MAC-RF for mothers; SDQ and SCAS-SAD for children) and some selected psychosocial variables of the survey created ad hoc for this study. As to the mentioned variables, data are normally distributed as verified by using the Shapiro–Wilk test.

Partial two-tailed correlations were performed between clinical parameters of asthma control and severity (GINA, ACT, and GINA therapeutic steps), mothers’ standardized questionnaires (GHQ-12 and MAC-RF), and selected psychosocial variables of the survey (e. g. worries for contagion, physical and psychological well-being). Correlations were controlled for the time passed from the end of the lockdown (May 18th, 2020) to the survey administration. In parallel, children’s medical measures were correlated with children’s psychosocial measures (SDQ and SAD factor of the SCAS) and selected indexes of the survey (e. g. worries for contagion, contacts with friends).

Regarding the clinical sample, a multiple linear regression model was performed to assess which variables were predictive of children’s psychological well-being. Children’s psychological well-being was used as a dependent variable, and children’s age and gender, time from the end of home-confinement, GINA scores, GINA therapeutic steps, children’s concerns for contagion and physical well-being, mothers’ psychological and physical well-being, and mothers’ total MAC-RF score, as independent variables. If considering the association between children’s adjustment problems and asthma severity literature reports inconsistent findings: some studies evidence the relation^33^, some others no^34^. Thus, it is an interesting aspect to explore. Moreover, literature reports that mothers with general psychological well-being are more willing to help their children manage their fears, thus influencing their psychological well-being^21^. Children’s psychological well-being variable consisted of a 3-point scale (0 = my general psychological well-being is better now than last year when I was going to school; 1 = my general psychological well-being is now the same as last year when I was going to school; 2 = my general well-being is now worse than last year when I was going to school). Higher scores indicated worse well-being, during the lockdown compared to the previous year. For all the analyses, a *p*-value < 0.05 was considered statistically significant. Statistical analysis was performed using the SPSS v22.0 software package (SPSS Inc., Chicago, USA).

## Results

Forty-five children (M_age_ = 10.67, SD_age_ = 2.29) with asthma were enrolled in this study (77.8% males). In the present sample, 80% of the children had well-controlled asthma, as reported by the GINA scores. As to asthma severity, assessed by GINA therapeutic steps, 44.5% had mild asthma while 55.6% had moderate asthma. Rhinitis was present in 60% of the sample. Also, their mothers (n = 45) were included in the study (M_age_ = 43.93, SD_age_ = 5.29). Most of the mothers had a high-school diploma (46.7%) and their professions were mainly intellectual (22.2%), executive (35.6%), and unskilled ones (e. g. housewives) (24.4%). (see Table 1).

**Table 1:** Demographics and descriptive characteristics of children and mothers of the clinical and control groups.

	Clinical sample (N = 45)		Control sample (N = 41)		t-test/χ^2^ test	*p*-value
Children	Mean	SD	Mean	SD		
Age	10.67	2.29	11.02	2.25	− .730	.467

		Percentages	Percentages			
Gender	Males	77.8%	68.3%		-.987	.327
	Females	22.2%	31.7%			
Asthma control—GINA scores	Well-controlled asthma	80%				
	Partially controlled	13.3%				
	Uncontrolled asthma	6.7%				
Asthma severity—GINA therapeutic steps	Mild asthma	44.5%				
	Moderate asthma	55.6%				
Rhinitis	Presence	60%				
	Absence	40%				

Mothers	Mean	SD	Mean	SD		
Age	43.93	5.29	45.61	5.42	− 1.449	.151

		Percentages	Percentages			
Schooling	Middle school diploma	20%	14.6%		-1.296	.198
	High school diploma	46.7%	34.1%			
	Graduated	31.1%	51.2%			
	Ph.D	2.2%	0%			
Occupation	Intellectual professions	22.2%	39%		.992	.324
	Executive professions	35.6%	24.4%			
	Business	13.3%	7.3%			
	Workers and craftsmen	4.4%	12.2%			
	Unskilled professions	24.4%	17.1%			

Student’s t-tests for continuous variables and χ^2^ tests for dichotomic variables to compare the two groups. All results are reported as mean (SD), or with the percentage where specified.

### Differences between clinical and control samples in standardized questionnaires and selected variables of the ad hoc survey

Most of the asthmatic children reported scores at the non-clinical range for SDQ (97.8%) and SCAS-SAD (73.3%).

As to the comparisons between asthmatic and control children’s groups, no differences were found in SDQ and SCAS-SAD scores.

Considering some selected variables of the ad hoc survey, asthmatic children reported higher levels of fear to be infected by Covid-19, if compared to healthy peers (p = 0.000), with medium effect size’s value. Results are shown in Table 2.

**Table 2:** Differences between clinical and control children samples’ scores in standardized questionnaires and in some selected variables of the ad hoc survey calculated using student’s T-test. Standard deviations are reported. Bold for *p*-values < .05. *Note: effect size’s r values: .00–.20 low, .30–.50 medium, .60–2.00 large.

	Clinical sample (N = 45)		Control sample (N = 41)		t	*p*	Effect size r*
	Mean	SD	Mean	SD			
SDQ							
**Standardized variables**							
Emotional symptoms	2.00	1.53	1.88	1.55	.366	.716	0.03
Conduct problems	2.29	1.54	2.59	1.65	− .858	.393	− .09
Hyperactivity/inattention	3.47	1.97	3.93	2.05	− 1.059	.292	− .11
Peer problems	1.36	1.56	1.10	1.35	.812	.419	.08
Prosocial behaviors	8.98	1.97	8.41	1.54	1.463	.147	.15
Total score	9.11	4.62	9.49	3.45	− .425	.672	− .04
Internalizing	2.80	2.24	2.66	1.79	.321	.749	.03
Externalizing	4.76	2.73	5.39	2.62	− 1.096	.276	− .11
SCAS							
Separation anxiety factor	5.31	3.704	4.68	3.357	.821	.414	.08
**Variables of the survey**							
** Concerns for contagion**	**1.91**	**.59**	**1.41**	**.54**	**4.012**	**.000**	**.40**
Contacts with friends	.91	.28	.90	.30	.137	.892	.01
Contacts with friends during home confinement	2.02	.85	2.27	.80	− 1.307	.195	− .14
Current contacts with friends	2.10	.86	2.11	.84	− .055	.957	− .01
Psychological well-being	2.58	.58	2.39	.70	1.351	.180	.14
Concerns about the resumptions of activities	2.62	1.11	2.51	1.14	.452	.652	.04

Regarding the general well-being of asthmatic children’s mothers measured with GHQ- 12, 51.1% of them reported psychological suffering and 31.1% possible need for intervention. As to Covid-19 related fears assessed with MAC-RF, 57.8% of the clinical sample’s mothers reported psychological suffering and 13.3% possible need for intervention.

According to the comparison between asthmatic children’s mothers and control group’s mothers, no differences were found in GHQ scores (*p* = 0.764). Instead, mothers of the clinical group reported more concerns about Covid-19 assessed by MAC-RF (Total MAC-RF score, *p* = 0.002), and stronger fears related to the body (*p* = 0.014), fears related to meaningful relationships (*p* = 0.004), and behavioral difficulties related to fears (*p* = 0.043), with medium effect size’s values.

In respect to the ad hoc survey selected variables, mothers of the clinical sample reported higher fears for their or their children’s contagion (respectively *p* = 0.012 and *p* = 0.000), and stronger concerns about resumptions of their children's activities (*p* = 0.000). Moreover, mothers of the clinical sample reported a general worsening of their psychological well-being through a retrospective evaluation, showing a trend towards statistical significance (*p* = 0.058). Effect sizes’ values for these variables are medium. Results are reported in Table 3.

**Table 3:** Differences between clinical and control mothers’ samples’ scores in standardized questionnaires and in some selected variables of the ad hoc survey, calculated using student’s T-test. Standard deviations are reported. Bold for *p*-values<.05.

	Clinical sample (N = 45)		Control sample (N = 41)		t	*p*	Effect size
	Mean	SD	Mean	SD			
**Standardized variables**							
GHQ-12							
Total score	18.00	4.59	17.76	2.76	.301	.764	.03
MAC-RF							
** Total score**	**14.04**	**5.54**	**9.98**	**5.99**	**3.271**	**.002**	**.33**
** Fears related to the body**	**3.56**	**2.30**	**2.37**	**2.05**	**2.517**	**.014**	**.26**
** Fears related to meaningful relationships**	**4.60**	**1.98**	**3.22**	**2.35**	**2.953**	**.004**	**.30**
Difficulties in cognitive monitoring of concerns	2.71	1.56	2.07	1.57	1.887	.063	.14
** Behavioral difficulties related to fear**	**3.18**	**1.93**	**2.32**	**1.95**	**2.051**	**.043**	**.21**
Variables of the survey							
** Concerns for contagion**	**1.93**	**.44**	**1.66**	**.53**	**2.587**	**.012**	**.26**
** Psychological well-being**	**2.62**	**.57**	**2.37**	**.66**	**1.921**	**.058**	**.19**
Physical well-being	2.38	.49	2.24	.48	1.266	.209	.14
** Worries for asthmatic child’s contagion**	**2.16**	**.52**	**1.68**	**.52**	**4.203**	**.000**	**.41**
Child’s psychological well-being (reported by mothers)	2.51	.54	2.41	.49	.850	.398	.09
Child’s physical well-being (reported by mothers)	2.22	.59	2.32	.47	− .811	.420	− .09
Communication	4.18	.68	4.17	.83	.043	.966	.01
Time spent with the child before home-confinement	7.24	4.97	6.34	3.73	.945	.347	.10
Time currently spent with the child	10.16	5.78	10.51	5.61	− .290	.773	− .03
** Concerns about the resumptions of child’s activities**	**3.31**	**.87**	**2.54**	**.97**	**3.879**	**.000**	**.38**

*Note: effect size’s r values: .00–.20 low, .30–.50 medium, .60–2.00 large.

### Association between asthmatic children’s medical measures and children and mothers’ standardized questionnaires and selected variables of the ad hoc survey

Regarding asthmatic children, the GINA scores correlated with their self-perceived physical well-being (r = 0.354, *p* = 0.025). Considering children’s standardized questionnaires, a significant positive correlation was found between the GINA scores and the “emotional symptoms” SDQ subscale (r = 0.299, *p* = 0.049) and the GINA scores and the SCAS-separation anxiety factor (r = 0.306, *p* = 0.043). ACT was negatively correlated with the SCAS-separation anxiety factor (r = − 0.473, *p* = 0.001).

No significant correlations emerged between the GINA therapeutic steps and children’s variables (both standardized questionnaires and ad hoc survey variables).

As to mothers, no significant correlations emerged.

### Predictors of asthmatic children’s psychological well-being

According to the literature, it was hypothesized that asthmatic children’s psychological well-being may be predicted by their age and gender, the severity and control of their asthma (GINA, GINA therapeutic steps), and their mothers’ psychological well-being. The multiple linear regression model (Table 4) showed that children’s psychological well-being was predicted by their physical well-being and their mothers’ psychological well-being. More specifically, a worsening of children’s physical and mothers’ psychological well-being was associated with a worsening of asthmatic children’s psychological well-being.

**Table 4:** Multiple linear regression model of asthmatic children’s psychological well-being (n = 45). Bold for *p*-values < .05.

	Children’s psychological well-being			
	*B* (95% CI)	Std. β	*t*	*p*
Intercept	− .232 (− 1.804, 1.339)		− .301	.766
Children’s age	.064 (− .005, .134)	.253	1.880	.069
Time from end of home-confinement	− .001 (− .010,.008)	− .031	− .234	.817
Child gender (2 = F)	− .085 (− .441, .272)	− .061	− .484	.631
GINA	− .120 (− .288, .047)	− .208	− 1.457	.154
GINA therapeutic steps	.049 (− .079, .176)	.104	.773	.445
Children’s concerns for contagion	− .092 (− .341, .157)	− .094	− .752	.457
**Mothers’ psychological well-being**	**.473 (.208, .738)**	**.467**	**3.630**	**.001**
MAC-RF total score	.006 (− .023, .035)	.060	.443	.660
**Children’s physical well-being**	**.630 (.353, .907)**	**.644**	**4.626**	**.000**
Mothers’ physical well-being	− .174 (− .498, .151)	− .146	− 1.088	.284
Model fit	F(10,44) = 3.519			
	*P* = .003			
Adj. R^2^	.364			

*B*, unstandardized beta; *std. β*, standardized beta; *CI*, confidence intervals.

## Discussion

The present paper focused on the assessment of asthmatic children and their mothers’ psychological well-being compared to that of their healthy peers, in the immediate period following lockdown due to the Covid-19 pandemic. The link between asthma and Covid-19 was still debated at the time of the survey’s administration^11^. It was unclear if asthma might be considered a risk factor for SARS-CoV-2 infection or some treatment used to control it might be protective against the infection^12,13^. Moreover, literature has not yet explored the psychological well-being of asthmatic children and their mothers in the immediate period following lockdown.

As for children, asthmatic patients did not differ from healthy peers in reporting psychological and separation anxiety symptoms, showing normative functioning in a post lockdown period. Literature comparing asthmatic and healthy children evidenced inconsistent results. The extreme reduction of in-person relationships or contact with peers might negatively affect children's perceived psychological well-being^5^. Considering specific asthma-related issues, on the one hand, mandatory home-staying might reduce the exposure to allergens and infections that frequently cause asthma attacks and could increase parental supervision for asthma symptom management^15^. On the other hand, being at home could more frequently expose children to indoor noxious environments^18^. Interestingly, asthmatic children in this study reported an overall good level of asthma control during the lockdown. This might impact positively their psychological symptoms. It’s noteworthy that the clinical sample in the present study did not include children with severe asthma.

As for mothers, most of them showed higher levels of psychological suffering. Although the present study did not include mothers’ psychological functioning evaluation before the Covid-19 outbreak, they reported a general perceived worsening of their psychological well-being in a post lockdown scenario. Moreover, they also had higher Covid-19-related fears, such as worries about their or their children’s contagion, in comparison with the control mothers. Given that mothers of the clinical and control samples had similar sociodemographic characteristics (e. g. age, schooling, occupation), we could hypothesize that the increase in Covid19-related fears could account for being a parent of an asthmatic child. Mothers’ worries were mainly focused on the possible consequences in case of Covid-19 infection for their children, because of their underlying chronic respiratory condition^16^. Future studies are needed to spread light on clinical mothers’ fears. It would be also interesting to understand if they are more worried about themselves or about eventually infecting their children.

Considering the relationship between asthma control and children’s psychological well-being, less controlled asthma (GINA scores) seemed to be associated with higher emotional symptoms (“emotional symptoms” SDQ subscale), such as sadness or worry, and separation anxiety symptoms (SCAS-separation anxiety factor) reported by children, although the majority of them showed an overall good level of asthma control. It is interesting to point out that the presence of an even lower level of asthma symptoms was associated with more intense fears of being alone (such as during night-time), of being in another room in the house or far from the parents (separation anxiety factor), in a reopening scenario after the lockdown^20^.

Finally, asthmatic children’s psychological well-being was associated with their physical well-being and their mothers’ psychological well-being. We can speculate that having good control of asthma symptoms is a protective factor for children in preventing the worsening of their psychological well-being during a pandemic characterized by severe pulmonary diseases. Moreover, mothers’ psychological health might be crucial in helping asthmatic children to express their psychological fatigue in Covid-19 pandemic scenarios. It might be hypothesized that mothers who are less worried and have good psychological well-being are more prone to manage asthma therapies for their children and to better contain their children’s possible worries^21^.

This study has some limitations. First, data of psychological functioning before the lockdown was not available, thus requiring retrospective information. Second, the small number of patients recruited makes it difficult to generalize the results. Moreover, the questionnaires’ administration period was quite long (3 months). Furthermore, Cronbach’s alfas for some SDQ subscales are low. However, similar values for some SDQ-children version subscales have been reported in previous studies with clinical samples (e.g. children with cleft lip/palate)^35,36^. Future studies need to address this point. This study has also some strengths: the sample’s characteristics, which include data from both children and mothers; the use of validated questionnaires. Besides, the administration of the survey after the quarantine period allowed to assess the short and long-term psychological effects of the restrictive policies, giving important information to develop adequate medical and psychological support programs.

In conclusion, the data suggest that mothers of asthmatic children could be more at risk of experiencing psychological fatigue, in a pandemic scenario. They had to face not only the stressors related to Covid-19 but also the triggers of managing children with respiratory diseases in such a pandemic. This study highlighted the importance of planning specific programs to help families with special needs. For example, it would be useful to provide educational and psychological support for asthmatic children’s mothers. Future investigation of the psychological functioning of asthmatic children’s fathers is recommended, as well as a deeper exploration of mothers’ worries in the pandemic scenario, especially whether their fears for contagion concern more themselves or their children.

## Data Availability

The datasets generated and analyzed during the current study are available from the corresponding author on reasonable request.
